# Structural Basis for Langerin Recognition of Diverse Pathogen and Mammalian Glycans through a Single Binding Site

**DOI:** 10.1016/j.jmb.2010.11.039

**Published:** 2011-01-28

**Authors:** Hadar Feinberg, Maureen E. Taylor, Nahid Razi, Ryan McBride, Yuriy A. Knirel, Sarah A. Graham, Kurt Drickamer, William I. Weis

**Affiliations:** 1 Department of Structural Biology, Stanford University School of Medicine, Stanford, CA, USA; 2 Department of Molecular and Cellular Physiology, Stanford University School of Medicine, Stanford, CA, USA; 3 Division of Molecular Biosciences, Department of Life Sciences, Imperial College, London SW7 2AZ, UK; 4 Glycan Array Synthesis Core-D, Consortium for Functional Glycomics, Department of Molecular Biology, The Scripps Research Institute, La Jolla, CA 92037, USA; 5 N. D. Zelinsky Institute of Organic Chemistry, Russian Academy of Sciences, 119991 Moscow, Russia

**Keywords:** CRD, carbohydrate-recognition domain, Man4, Manα1–3(Manα1–6)Manα1–6Man, Man5, Manα1–3(Manα1–6)Manα1–6(Manα1–3)Man, PDB, Protein Data Bank, laminaritriose, Glcβ1–3Glcβ1–3Glc, PEG, polyethylene glycol, langerin, C-type lectin, carbohydrate recognition

## Abstract

Langerin mediates the carbohydrate-dependent uptake of pathogens by Langerhans cells in the first step of antigen presentation to the adaptive immune system. Langerin binds to an unusually diverse number of endogenous and pathogenic cell surface carbohydrates, including mannose-containing O-specific polysaccharides derived from bacterial lipopolysaccharides identified here by probing a microarray of bacterial polysaccharides. Crystal structures of the carbohydrate-recognition domain from human langerin bound to a series of oligomannose compounds, the blood group B antigen, and a fragment of β-glucan reveal binding to mannose, fucose, and glucose residues by Ca^2+^ coordination of vicinal hydroxyl groups with similar stereochemistry. Oligomannose compounds bind through a single mannose residue, with no other mannose residues contacting the protein directly. There is no evidence for a second Ca^2+^-independent binding site. Likewise, a β-glucan fragment, Glcβ1–3Glcβ1–3Glc, binds to langerin through the interaction of a single glucose residue with the Ca^2+^ site. The fucose moiety of the blood group B trisaccharide Galα1–3(Fucα1–2)Gal also binds to the Ca^2+^ site, and selective binding to this glycan compared to other fucose-containing oligosaccharides results from additional favorable interactions of the nonreducing terminal galactose, as well as of the fucose residue. Surprisingly, the equatorial 3-OH group and the axial 4-OH group of the galactose residue in 6SO_4_–Galβ1–4GlcNAc also coordinate Ca^2+^, a heretofore unobserved mode of galactose binding in a C-type carbohydrate-recognition domain bearing the Glu-Pro-Asn signature motif characteristic of mannose binding sites. Salt bridges between the sulfate group and two lysine residues appear to compensate for the nonoptimal binding of galactose at this site.

## Introduction

Langerhans cells found in the epidermis of the skin and in the epithelium of mucosal tissues take up and process antigens from invading pathogens for presentation to T cells and thus have a critical role in the adaptive immune response. Langerhans cells are characterized by the presence of langerin (CD207), a C-type lectin that can act as a pathogen receptor by binding to surface glycoconjugates of microorganisms. Langerin binds to fungi, including *Malassezia furfur* and a number of *Candida* and *Saccharomyces* species;^1,2^ to mycobacteria, including *Mycobacterium leprae*;^3^ and to viruses such as human immunodeficiency virus and herpes simplex virus 2.^4,5^ Langerin protects against human immunodeficiency virus infection by binding and internalizing the virus to Birbeck granules for degradation.^4,5^

Langerin has an extracellular region that contains (1) a C-type carbohydrate-recognition domain (CRD) that binds sugars and (2) a neck region that mediates the formation of trimers.^6^ Langerin recognizes pathogens by binding to high-mannose structures on viral envelope glycoproteins and to mannan and β-glucan structures on fungal cell walls.^1,2,5,7^ Glycan array analysis has shown that langerin also binds to oligosaccharides bearing the blood group B antigen Galα1–3(Fucα1–2)Gal and to 6-sulfated galactosides and high-mannose N-linked oligosaccharides.^2,7,8^ As in other C-type lectins, the CRD of langerin contains a conserved Ca^2+^ binding site that binds to carbohydrates.^9^ The Ca^2+^ forms direct coordination bonds with vicinal hydroxyl groups on the sugars, which also form hydrogen bonds with amino acid side chains that are also Ca^2+^ ligands.^10^

C-type lectins broadly bind either to ligands containing a pair of vicinal equatorial hydroxyl groups in the same stereochemistry as the 3-OH and 4-OH groups of d-mannose or to sugars bearing vicinal hydroxyl groups equivalent to the equatorial 3-OH group and the axial 4-OH group of d-galactose. C-type CRDs recognizing mannose-type ligands, including d-mannose, d-GlcNAc, d-glucose, and l-fucose, have a signature sequence motif Glu-Pro-Asn (EPN) on one side of the conserved Ca^2+^ site, whereas the equivalent residues in CRDs that bind to galactose-type ligands have a signature sequence motif Gln-Pro-Asp (QPD).^11^ Although langerin binding to many of the ligands identified to date is consistent with the presence of the EPN signature motif, there are important aspects of ligand binding specificity that remain to be explained structurally, including selectivity for a specific subset of ligands containing mannose, fucose, or glucose. In addition, recognition of 6-sulfated galactosides is unexpected because of the presence of the EPN motif.

Here we report the identification of additional glycan ligands for langerin through screening of a microarray of bacterial lipopolysaccharides and high-resolution crystal structures of the langerin CRD bound to carbohydrates representing a spectrum of ligands. The structures explain how langerin can bind to such a diverse set of ligands, as well as why some glycans are not recognized by this protein.

## Results and Discussion

### Novel mannose-containing ligands for langerin

**Fig. 1:**
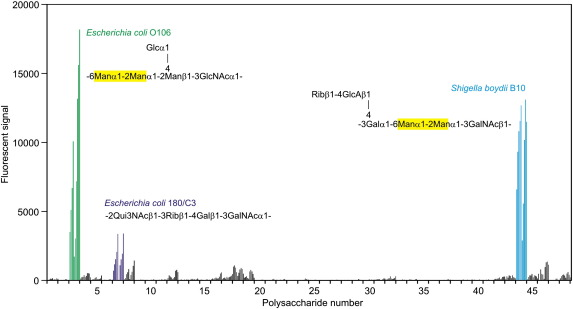
Binding of langerin to bacterial polysaccharide microarray. Each O-specific polysaccharide + core released from a bacterial lipopolysaccharide was printed in 10 versions. The first five versions were printed directly without modification, and the second five versions were derivatized with a disubstituted oxamine linker prior to printing. Each version was printed at 0.03 mg/mL, 0.06 mg/mL, 0.125 mg/mL, 0.25 mg/mL, and 0.5 mg/mL. The data shown represent the averages of six replicate spots for each version. A complete list of the bacterial sources of the lipopolysaccharides and their full O-specific polysaccharide core structures is provided in Supplementary Table 1.

A previous glycan array analysis has revealed three classes of high-affinity ligands for langerin: high-mannose N-linked oligosaccharides, the fucose-containing blood group B trisaccharide, and glycans terminating in sulfated galactose residues.^7^ Studies on binding to fungi and glycans derived from them have identified both mannans and β-glucans as ligands.^1^ Given this broad and structurally diverse spectrum of ligands, the possibility that there are other classes of ligands was investigated using newly developed microarray technology to screen a selection of glycans derived from bacterial lipopolysaccharides (R.M., N.R., O. Berger, Y.A.K., and J. C. Paulson, in preparation). From a panel of 48 test ligands, two of the polysaccharides give clear and consistent positive signals (Fig. 1). In both of these cases, there are Manα1–2Man units in the backbone repeat units of the polysaccharides, both derivatized at the 6-position on the nonreducing side. No other monosaccharide residues in these two polysaccharides would have adjacent free hydroxyl groups available, which would be required for ligation to the Ca^2+^-centered binding site in the langerin CRD. In addition, none of the other polysaccharides on the array contains the Manα1–2Man unit, suggesting that internal Manα1–2Man disaccharides within an oligosaccharide polymer are specific targets for langerin binding. These results indicate that potential mannose-containing ligands for langerin include bacterial surface polysaccharides, as well as mannan-type structures on yeasts and high-mannose oligosaccharides on viruses.

### Binding to high-mannose oligosaccharides

**Fig. 2:**
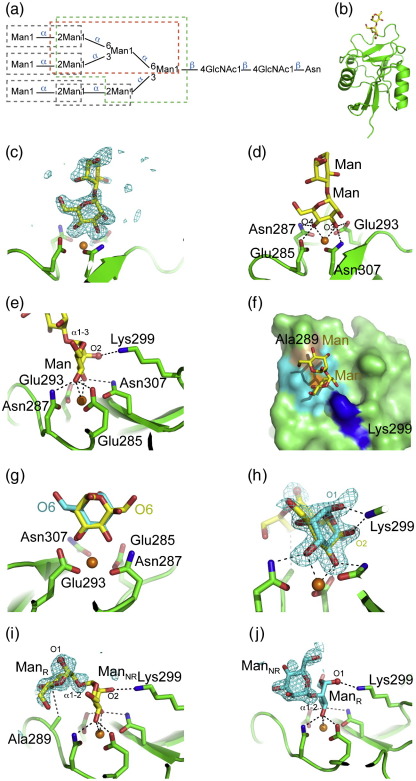
Structure of the CRD of langerin bound to high-mannose carbohydrates. In all panels, the protein is shown in green cartoon representation, with some selected side chains shown in stick representation. Ca^2+^ is shown as an orange sphere. Carbohydrates are shown in stick representation. Oxygen atoms are shown in red, and nitrogen atoms are shown in blue. In (e), (i), and (j), oxygen atoms that would be linked to other sugars in larger N-linked oligosaccharides are shown as red spheres. (a) Diagram of an N-linked high-mannose oligosaccharide showing the linkages in Man5 (green broken box), Man4 (red broken box), and Manα1–2Man (gray broken boxes). (b) Overall secondary structure of langerin bound to the Manα1–3Man disaccharide from Man5. (c) *F*_o_ − *F*_c_ electron density (cyan) for the Manα1–3Man disaccharide that is visible in copy A, calculated by omitting the mannose residues from the model contoured at 2.7σ. (d and e) Man5 bound at the Ca^2+^ binding site. (f) Langerin carbohydrate-binding surface. Langerin CRD bound to Manα1–2Man. The protein is shown in surface representation: residues not interacting with the carbohydrate (green), Ca^2+^ ligands (cyan), Lys299 (blue), and other residues interacting with the sugar (light brown). Ca^2+^ is shown in orange. (g) Two binding modes for the mannose residue of Manα1–2Man at the Ca^2+^ binding site in copy A. (h) Two binding modes for the mannose residue of Manα1–2Man at the Ca^2+^ binding site in copy B, superimposed on an *F*_o_ − *F*_c_ omit map contoured at 3σ. (i and j) Alternative binding modes for Manα1–2Man. The reducing Man residue of Manα1–2Man is marked Man_R_, and the nonreducing Man residue of Manα1–2Man is marked Man_NR_. The second mannose residue (i.e., the residue not directly bound to Ca^2+^) is shown superimposed on an *F*_o_ − *F*_c_ omit map contoured at 2.5σ. (i) The first conformation of the disaccharide seen in copy C. (j) The alternative conformation observed in copy B.

In order to investigate the potential modes of binding of langerin to both eukaryotic and prokaryotic mannose-containing sugars, we determined the crystal structures for the monomeric full-length langerin CRD bound to various mannose-containing oligosaccharides. The crystals used for these complexes and for all complexes described in this article contain four copies of the CRD in the asymmetric unit, referred to as copies A, B, C, and D. In the first set of experiments, binding to both eukaryotic and prokaryotic mannose-containing oligosaccharides was investigated. The first two oligosaccharides investigated represent substructures of the eukaryotic mannose-containing ligands: Manα1–3(Manα1–6)Manα1–6Man (Man4) and Manα1–3(Manα1–6)Manα1–6(Manα1–3)Man (Man5) (Fig. 2a). For both ligand complexes, electron density was observed in the conserved Ca^2+^ site in each of the four crystallographically independent copies of the CRD and was fitted with a mannose (Fig. 2b and c). In each copy, mannose binds with the 3-OH group interacting with the Ca^2+^ ligands Glu293 and Asn307, while the 4-OH group interacts with Glu285 and Asn287, which are part of the EPN motif (Fig. 2d). In this orientation, the axial 2-OH group forms a hydrogen bond with Lys299 (Fig. 2e).

In all of the Man4 complexes, as well as in most of the copies of the Man5 complex, only a single-mannose residue is visible, and it cannot be determined whether this corresponds to a particular mannose residue present in these ligands. However, in one copy of the CRD–Man5 complex, there is additional electron density that is best interpreted as an α1–3-linked mannose (Fig. 2c). Moreover, there is weak density emerging from the anomeric oxygen of this second sugar. These observations indicate that the mannose residue in the primary binding site corresponds to the outer α1–3-linked mannose residue at the nonreducing end of the Man5 oligosaccharide, consistent with the suggestion that langerin can bind to mannose residues at the nonreducing termini of branched structures. At least some of the enhanced binding seen for larger oligosaccharides on the glycan array such as Man5, compared to a simple branched Man3 structure, may reflect the presence of multiple terminal residues, each of which can bind in the primary binding site.^7^

Insight into the mechanism of the binding of langerin to glycans containing Manα1–2Man groups, found at the termini of larger branched high-mannose eukaryotic glycans and at internal positions in the O-specific polysaccharide chains of bacterial lipopolysaccharides, was obtained from the crystal structure of the CRD complexed with a Manα1–2Man disaccharide (Fig. 2f). Electron density for both sugars in the disaccharide is seen in two copies of the CRD, whereas only a single-mannose residue is observed in the other two. The disaccharide adopts the preferred conformation seen in other crystal structures.^12,13^ Unlike in the Man4 and Man5 structures, two orientations for the mannose bound in the Ca^2+^ site are observed, each at approximately 50% occupancy, in each of the four crystallographically independent copies of the Man2–CRD complex (Fig. 2g and h). One orientation is the same as that observed for the terminal mannose residue in the Man4 and Man5 structures, in which the 3-OH group coordinates Ca^2+^ and forms hydrogen bonds with Glu293 and Asn307, the 4-OH group coordinates Ca^2+^ and forms hydrogen bonds with Glu285 and Asn287, and the 2-OH group forms a hydrogen bond with Lys299. When the full disaccharide is visible in this orientation, the residue at the nonreducing end is in the primary binding site, and the 4-C of the mannose residue at the reducing end packs against Ala289 (Fig. 2f and i). In the second orientation, the sugar ring is rotated about an axis perpendicular to the C3–C4 bond, so that the partners of the 3-OH and 4-OH groups are reversed, and the oxygen attached to the anomeric carbon forms a hydrogen bond with Lys299 (Fig. 2g and h). In this orientation, when the disaccharide is visible, the reducing end residue is the one ligated to Ca^2+^, and the second residue does not contact the protein (Fig. 2j).

The structural data indicate that Manα1–2Man groups found at the termini of high-mannose N-linked glycans may bind in both orientations observed in the crystal structure. In the first conformation (Fig. 2i), the anomeric carbon at the reducing end of Manα1–2Man points away from the protein surface, so there is room to accommodate the internal sugars to which the Manα1–2Man units are attached, regardless of whether the linkage is α1–6, α1–3, or α1–2 (Fig. 2a). The second orientation, in which the reducing mannose is bound at the Ca^2+^ site (Fig. 2j), would also be able to accommodate terminal Manα1-2Man linked α1–6 or α1–3 to internal sugars with no steric clash. However, unless the position of Lys299 is changed, the terminal Manα1–2Man-linked α1–2 on the third branch could not bind, and we see no evidence for an alternative position of this lysine or electron density for mannose corresponding to this arrangement. These observations indicate that there may be multiple modes of binding to the terminal disaccharide of high-mannose N-linked glycans; however, given the extra contacts formed by the reducing mannose in Fig. 2i, it seems likely that this orientation is the preferred mode of binding to Manα1–2Man-containing compounds.

The structures also suggest how the internal Manα1–2Man units found in the two strongly interacting glycans derived from bacterial lipopolysaccharides (Fig. 1) may bind. In the first conformation (Fig. 2i), the chain can be readily extended from the reducing end. At the other end of the disaccharide, glycans in which the 6 position of the mannose bound at the Ca^2+^ site is on the reducing side of an α1–6-linked sugar can also be accommodated, since the 6-OH group of this mannose points away from the protein surface. In the alternative conformation (Fig. 2j), the 6 position of the nonreducing mannose is away from the protein surface, allowing elongation from this end, and an α1–3 linkage of the Ca^2+^-ligated mannose to GalNAc, as in polysaccharide 44, could also be accommodated. This mode of binding would not be possible, however, for polysaccharide 3 as the α1–2 linkages formed by the first two mannose residues may be sterically inaccessible, as noted above for high-mannose N-linked glycans, and the 4-OH group of the third mannose is linked to another sugar and therefore cannot bind to Ca^2+^. Thus, as in the case of the high-mannose structures, the binding mode represented in Fig. 2i would seem to be favored over that of Fig. 2j due to the more facile accommodation of various linkages and the extra contacts formed with the protein.

Collectively, the structural data indicate that the interaction of langerin with mannose-containing oligosaccharides involves significant contacts primarily with a single mannose residue at the conserved Ca^2+^ site and, in the binding mode shown in Fig. 2i, an additional contact with the second α1–2-linked mannose. There are also water-mediated contacts, as observed in other protein carbohydrate complexes.^14^ Thus, in contrast to another dendritic cell receptor that recognizes high-mannose oligosaccharides, DC-SIGN (*d*endritic-*c*ell-*s*pecific *i*ntercellular adhesion-molecule-3-*g*rabbing *n*onintegrin),^15^ langerin has only a small extended binding site that contacts other sugar residues in these oligosaccharides. The structural data show no evidence for a proposed elongated site found by the modeling of Manα1–2Manα1–2Man and Manα1–2Manα1–3Man in the binding site.^16^ Given the 41.5-Å spacing between the binding sites of the langerin trimer,^7^ it is also unlikely that multiple termini from these oligosaccharides could simultaneously bind to multiple CRDs in a trimer. Instead, enhanced binding of the langerin CRD to high-mannose oligosaccharides relative to mannose may be a statistical effect of having multiple available mannose residues, as reported for DC-SIGN.^17^ Modeling indicates that, as observed in Man4 binding to DC-SIGN, an internal mannose, rather than a nonreducing terminal mannose, could be accommodated at the conserved Ca^2+^ site,^16^ which would increase the number of potential binding modes.

A second Ca^2+^-independent site for mannose was reported in a structure of langerin bound to mannose monosaccharide.^9^ We see no evidence for mannose in this site in any of the 12 crystallographically independent views of langerin binding to the Manα1–2Man, Man4, or Man5 oligosaccharides. In a previous report, this site was present in only two of the four copies in the asymmetric unit, and inspection of electron density maps made from deposited coordinates and structure factors indicates that the electron density in this site was misidentified as a sugar. Instead, it appears to be a tryptophan residue from the C-terminal affinity purification tag of a neighboring molecule in the lattice, as it is also present in the unliganded structure. The CRD construct used here contains no affinity tags, and this density is absent. The authors of the previous study have confirmed that the density was indeed misidentified, and the deposited coordinates have been updated [A. Skerra, personal communication; Protein Data Bank (PDB) entries 3P7G, 3P7H, and 3P7F]. Note that this region of the protein is a flexible loop, and there appears to be some weak electron density corresponding to an alternative loop conformation;^7^ it was modeled here as such a mixture in some of the copies in the asymmetric units.

### Structure of langerin bound to the blood group B trisaccharide

The primary fucose-containing ligands for langerin identified by glycan array analysis contain the blood group B epitope Galα1–3(Fucα1–2)Gal. This oligosaccharide is found in several pathogenic bacteria such as *Helicobacter pylori* and *Escherichia coli*. In the case of *E. coli* strain O86, it has been suggested that binding of galectins in the gut to the blood group B epitope may result in pathogen neutralization,^18^ but the binding of langerin to the same epitope suggests that it may also participate in the defense against some strains of bacteria. In contrast to a number of other C-type CRDs, langerin does not bind Lewis^a^ and Lewis^x^ trisaccharides. In order to investigate the basis for this binding specificity, we determined the structure of the langerin CRD bound to the blood group B trisaccharide.

**Fig. 3:**
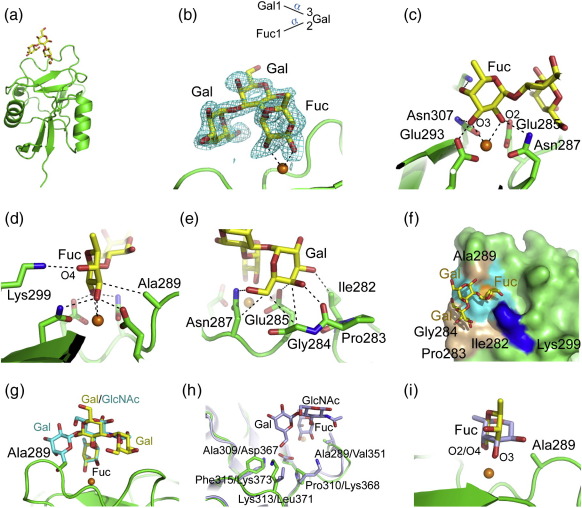
Structure of the CRD of langerin bound to the blood group B trisaccharide. The color scheme is the same as that described in Fig. 2. (a) Overall structure of protomer A bound to the trisaccharide. (b) Top: Diagram of the blood group B trisaccharide showing linkages between the sugars. Bottom: *F*_o_ − *F*_c_ electron density (3.0σ; cyan), calculated by omitting the trisaccharide model. (c and d) Fucose moiety bound at the Ca^2+^ binding site. (e) Terminal galactose moiety of the blood group B trisaccharide in the extended binding site. (f) Langerin carbohydrate-binding surface. Langerin CRD bound to the blood group B trisaccharide. The protein is shown in surface representation: residues not interacting with the carbohydrate (green), Ca^2+^ ligands (cyan), Lys299 (blue), and other residues interacting with the sugar (light brown). Ca^2+^ is shown in orange. (g) Model for Lewis^x^ binding through hydroxyl groups 2 and 3 of fucose. The fucose moiety of Lewis^x^ bound to DC-SIGN^19^ (PDB entry 1SL5) was superimposed on the fucose of the blood group B trisaccharide. Carbon atoms in the Lewis^x^ trisaccharide are shown in cyan. (h) Model for Lewis^x^ binding through hydroxyl groups 3 and 4 of fucose. The CRD of DC-SIGN bound to lacto-*N*-fucopentaose III (PDB entry 1SL5) was superimposed on the CRD of langerin. For simplicity, only the Lewis^x^ moiety of lacto-*N*-fucopentaose III is shown (light blue). (i) Comparison of the two potential modes for fucose binding to the Ca^2+^ site. Fucose from the structure of langerin bound to the blood group B trisaccharide is shown in yellow, and fucose from the superposition described in (g) is shown in light blue.

The bound blood group B trisaccharide was found to have the same orientation in all four CRDs in the asymmetric unit of the cocrystals. The fucose moiety binds to the Ca^2+^ site, with the equatorial 2-OH and 3-OH groups coordinating Ca^2+^ (Fig. 3a–c). In addition to the four Ca^2+^ ligands that form hydrogen bonds to the 2-OH and 3-OH groups of fucose, the 4-OH group forms a hydrogen bond with Lys299, and the ring 3-C packs against the side chain of Ala289 (Fig. 3c and d). The central galactose residue is positioned away from the protein, but the nonreducing galactose residue packs against C^α^ of Gly284 and the side chains of Ile282, Glu285, and Asn287. The 4-OH group of this galactose also forms a hydrogen bond with the backbone carbonyl oxygen of Pro283, and the 6-OH group is hydrogen bonded to the side-chain amide group of Asn287 (Fig. 3e and f). Since Asn287 also donates a hydrogen bond to the bound fucose, this residue bridges the fucose and galactose moieties (Fig. 3c and e). The 2-OH group of the nonreducing terminal galactose points away from the protein surface, explaining why the blood group A trisaccharide GalNAcα1–3(Fucα1–2)Gal can bind to langerin, albeit more weakly than the blood group B trisaccharide.

There are multiple instances of fucose-containing ligands that bind to C-type CRDs with the EPN signature, but two very different orientations of fucose residues in the primary binding site have been observed. Fucose monosaccharide is bound to the relatively open binding site in the CRD of rat serum mannose-binding protein with the 2-OH and 3-OH groups coordinated to Ca^2^^+^; thus, in the absence of other contacts with the protein, this configuration appears to be energetically favored.^20,21^ However, in oligosaccharides, the 3-OH and 4-OH groups of a fucose residue in the primary binding site are often coordinated to Ca^2+^. For example, the latter configuration is seen for ligands that contain the Lewis^x^ group Galβ1–4(Fucα1–3)GlcNAc bound to selectins^22^ or to DC-SIGN.^19^ Binding of the fucose residues in this orientation results in favorable contacts with both fucose and galactose residues, while the different configuration of the blood group B trisaccharide and the different arrangement of residues in the region around the primary binding site in langerin result in a different set of favorable contacts when the fucose residue in the blood group B trisaccharide is coordinated through the 2-OH and 3-OH groups.

Unlike DC-SIGN, langerin does not bind the Lewis^x^ and Lewis^a^ (Galβ1–3(Fucα1–4)GlcNAc) trisaccharides.^1,5^ The Lewis^x^ trisaccharide has a well-defined conformation, which enables modeling to probe why it cannot bind to langerin. Lewis^x^ binding to the langerin CRD was modeled in two possible ways. In one case, the fucose of Lewis^x^ was superimposed onto the fucose residue of the blood group B trisaccharide, with the 2-OH and 3-OH groups coordinating Ca^2+^ (Fig. 3g). In this case, the central GlcNAc of Lewis^x^ would be positioned away from the protein similarly to the central galactose residue of the blood group B trisaccharide. However, because the fucose is linked to the 3 position of GlcNAc rather than to the 2 position, the GlcNAc is rotated about 60° with respect to the reducing galactose residue in the blood group B trisaccharide. This change places the β-linked galactose residue in Lewis^x^ on the opposite side of the central sugar relative to the position of galactose in the blood group B trisaccharide. It would thus clash with Ala289, which is part of a rigid loop in langerin and other C-type CRDs (Fig. 3g). In the second model, the CRDs of DC-SIGN and langerin were superimposed so that the 3-OH and 4-OH groups of the fucose residue coordinate Ca^2+^ (Fig. 3h and i). In this case, there are no clashes with the CRD, but key residues that stabilize DC-SIGN binding to Lewis^x^ are absent in langerin, since residues Asp367, Lys368, Leu371, and Lys373 of DC-SIGN are replaced by Ala309, Pro310, Lys313, and Phe315 in langerin (Fig. 3h).

### β-Glucan binding

**Fig. 4:**
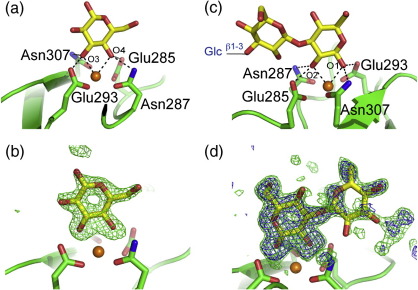
Structure of the CRD of langerin bound to laminaritriose. The color scheme is the same as that described in Fig. 2. (a) Nonreducing glucose residue bound to monomer C. (b) *F*_o_ − *F*_c_ electron density map (green), calculated by omitting the sugars (3.0σ) showing laminaritriose monomer C. (c) The reducing and central glucose residues visible in monomer B. (d) *F*_o_ − *F*_c_ electron density omit maps of langerin CRD bound to laminaritriose monomer B. Map contours at 3.0σ and 2.2σ are shown in blue and green, respectively.

Langerin binding to mannans and β-glucans that are components of fungal cell walls has been documented.^1^ The structures described above suggest a mechanism for the binding of Manα1–2Man units in the main chains or branches of mannans. The interaction with β-glucans was probed using a complex of Glcβ1–3Glcβ1–3Glc (laminaritriose) bound to the langerin CRD. In one copy of the CRD, a single-glucose residue representing the nonreducing end of the trisaccharide is visible, with its 3-OH and 4-OH groups coordinating Ca^2+^ and forming hydrogen bonds with Ca^2+^ ligands, as previously seen for the reducing glucose residue in maltose (Glcβ1–4Glc)^9^ (Fig. 4a and b). However, no additional interactions with the other two sugar residues are observed. In β1–6-linked glucans, there would be multiple residues that could bind in this mode, although only the nonreducing termini of β1–3 glucans would be able to bind. In another copy, a Glcβ1–3Glc disaccharide is visible, with the reducing end of the trisaccharide binding to Ca^2+^ through the equatorial 1-OH and 2-OH groups (Fig. 4c and d), indicating that langerin could bind to either end of a free glucan. The observed β1–3 linkage falls in the broad conformational minimum observed in databases of glycan structures.^13^

### Binding to glycans bearing 6-sulfated galactose

In contrast to the other ligands identified by screening on synthetic and pathogen glycan arrays, oligosaccharides bearing terminal 6SO_4_–Gal residues are not found on pathogen surfaces. However, binding to such structures has been suggested to mediate interaction with endogenous sulfated ligands such as keratan sulfate, which contains the 6SO_4_–Galβ1–4GlcNAc repeating unit.^2^ Langerin is unique among receptors with C-type CRDs because of its ability to bind both mannose/fucose-type ligands and a galactose-based ligand. The structural basis for this unusual binding was investigated by determining the structure of the CRD with the bound 6SO_4_–Galβ1–4GlcNAc ligand.

**Fig. 5:**
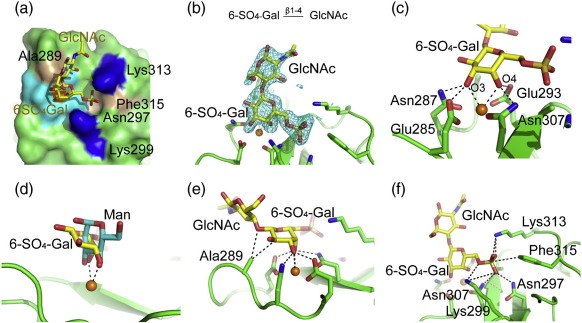
Structure of the CRD of langerin bound to 6SO_4_–Gal–GlcNAc. The color scheme is the same as that described in Fig. 2. (a) Langerin carbohydrate-binding surface. Langerin CRD bound to 6SO_4_–Galβ1–4GlcNAc. The protein is shown in surface representation: residues not interacting with the carbohydrate (green), Ca^2+^ ligands (cyan), Lys299 and Lys313 (blue), and other residues interacting with the sugar (light brown). Ca^2+^ is with in orange. (b) Top: Chemical structure of 6SO_4_–Galβ1–4GlcNAc. Bottom: *F*_o_ − *F*_c_ omit map (3.0σ) of 6SO_4_–Galβ1–4GlcNAc (cyan). (c) 6SO_4_–Gal at the Ca^2+^ binding site. (d) Comparison of mannose binding (cyan) and 6SO_4_–Gal binding (yellow) at the Ca^2+^ site in langerin. The CRDs observed in the Man4 and 6SO_4_–Galβ1–4GlcNAc complexes were superimposed in order to position the mannose residue on the 6SO_4_–Galβ1–4GlcNAc structure. (e) Side view of the Ca^2+^ site bound to 6SO_4_–Galβ1–4GlcNAc. (f) The position of the sulfate moiety of 6SO_4_–Galβ1–4GlcNAc in the binding site.

Electron density in all four copies of the CRD clearly shows both 6SO_4_–Gal and GlcNAc residues (Fig. 5a and b). In spite of the fact that langerin binds poorly to galactose compared to mannose and fucose,^6^ the galactose residue is bound in the Ca^2+^ site (Fig. 5a and c). The Ca^2+^ ligands Glu293 and Asn307 form hydrogen bonds with the axial 4-OH group of 6SO_4_–Gal, and Glu285 and Asn287 coordinate Ca^2+^ and form hydrogen bonds with the equatorial 3-OH group of 6SO_4_–Gal. The equatorial/axial geometry of these two hydroxyl groups tilts the galactose pyranose ring relative to mannose (Fig. 5d and e) so that it packs against Ala289. The SO_4_ group forms salt bridges with Lys299 and Lys313, consistent with previously published results showing that Lys299Ala or Lys313Ala mutations abolish binding to the disaccharide^2^ (Fig. 5f). The charge–charge interactions between these lysine residues and the sulfate group must compensate for the presumably nonoptimal Ca^2+^ ligation of galactose at a site with an EPN signature because langerin does not bind to nonsulfated galactosides.^2,6^

Previous studies showed binding of langerin to Lewis^x^-type carbohydrates containing 6-sulfated galactose.^2,8^ Since langerin does not bind Lewis^x^, high-affinity binding to 6SO_4_–Lewis^x^ likely arises from interactions of the sulfated galactose similar to those described here for 6SO_4_–Galβ1–4GlcNAc. The structure also explains why langerin does not bind to 3-sulfated galactose groups,^2,7,8^ since the 3-OH group is involved in Ca^2+^ coordination and hydrogen-bonding interactions in the conserved site.

## Conclusion

Langerin combines the features of several other C-type lectins. The organization of the trimer suggests that the multiple binding sites are widely spaced and probably rigidly positioned in a manner reminiscent of mannose-binding protein.^7^ However, although the binding site in each CRD is relatively open as in mannose-binding proteins, the glycan array results reported here and elsewhere indicate that langerin shows preferential binding to specific ligands rather than broad specificity resulting from the binding of just terminal sugar residues, as seen for mannose-binding protein. The structures of the langerin–ligand complexes reveal that this preferential binding results from a small number of favorable contacts with portions of selected oligosaccharide ligands beyond the monosaccharides sitting in the primary binding site (Figs. 2f, 3f, and 5a). In this respect, the binding site has some of the features of the binding site in DC-SIGN, although secondary contacts are less extensive. The structural analysis also shows that the primary binding site is able to bind both mannose-type and galactose-type ligands, but the absolute requirement for the sulfation of the galactose residue is explained by direct interactions with a positively charged region of the extended binding site. The subtlety of the secondary interactions that determine the binding selectivity of langerin illustrates the difficulty of predicting binding specificity from a comparative analysis of even closely related CRDs.

## Materials and Methods

### Carbohydrates

6SO_4_–Galβ1–4GlcNAc was purchased from Carbosynth, and Manα1–2Man was purchased from Sigma. All other sugars were purchased from V-labs, Inc.

### Protein production and labeling

The full-length CRD of human langerin was expressed in *E. coli* and purified by affinity chromatography on mannose Sepharose, as described previously.^6^ The CRD construct used contains residues 193–328 of human langerin similar to the one used in a previous crystallographic study,^9^ but it does not have any appended tag for purification. A stable trimeric fragment of the extracellular domain containing part of the neck region, as well as the CRD (designated truncated langerin), was also produced in *E. coli* and labelled with fluorescein, as described previously.^7^

### Glycan microarray analysis

Details of the bacterial polysaccharide microarray preparation will be described elsewhere (R.M., N.R., O. Berger, Y.A.K., and J. C. Paulson, in preparation)†. Lipid A was removed by mild acid hydrolysis, and the remaining O-specific polysaccharide + core structures were either covalently attached (through amino groups in the inner core) directly to *N*-hydroxysuccinimide-activated glass slides or derivatized at the reducing Kdo residue with 2-aminoethyl-(*N*-methyl)-hydroxylamine before spotting. The microarray was screened with fluorescein-labelled truncated langerin following the standard procedure of Core H of the Consortium for Functional Glycomics (protocol cfgPTC_243‡).

### Crystallization and data collection

Crystals of langerin CRD–carbohydrate complexes were obtained by hanging-drop vapor diffusion, using a mixture of 1.5 μL of protein–carbohydrate complex and 0.75 μL of reservoir solution in a drop. All crystals were grown at 22 °C. Crystals of langerin CRD bound to the blood group B trisaccharide and mannose-containing oligosaccharides were grown from a protein solution comprising 5 mg/mL langerin CRD, 2.5 mM CaCl_2_, 10 mM Tris–Cl (pH 8.0), and 25 mM NaCl. Carbohydrates were added to the protein solution prior to crystallization to a final concentration of 5 mM blood group B trisaccharide, 10 mM Man5 and Man4 oligosaccharides, or 30 mM Manα1–2Man. The complexes with 6SO_4_–Galβ1–4GlcNAc and laminaritriose were crystallized from a solution containing 2.5 mg/mL langerin CRD, 1.25 mM CaCl_2_, 5 mM Tris (pH 8.0), and 12.5 mM NaCl, with 6SO_4_–Galβ1–4GlcNAc or laminaritriose added to final concentrations of 7.5 mM and 15 mM, respectively. The reservoir solution contained 0.1 M Hepes (pH 7.0), 0.25 M MgCl_2_, and 20%, 25%, or 31% polyethylene glycol (PEG) 4000 for crystals containing blood group B and Man5 and Man4 oligosaccharides, respectively. For the crystallization of langerin CRD bound to Manα1–2Man, 6SO_4_–Galβ1–4GlcNAc, or laminaritriose, the reservoir solution contained 0.1 M Hepes (pH 7.0), 0.1 M MgCl_2_, and 25%, 25%, or 20% PEG 4000.

Crystals of langerin CRD complexed with Man5 oligosaccharide transferred to a solution containing all components of the crystallization drop apart from the protein, but with a PEG 4000 concentration of 34%, were frozen in liquid nitrogen for data collection. Other crystals were transferred to perfluoropolyether (PFO-XR75; Lancaster Synthesis) before being frozen in liquid nitrogen for data collection. Diffraction data were measured at 100 K on beamline 11-1 of the Stanford Synchrotron Radiation Lightsource. Data were processed with MOSFLM and SCALA,^23^ and are summarized in Table 1.

### Structure determination

All crystals belonged to space group *P*4_2_ with unit cell parameters very similar to those published for the langerin CRD,^9^ allowing structure solution directly by rigid-body refinement. The crystallographic asymmetric unit contains four copies of the CRD. Because of the ambiguity in assigning the direction of the *c*-axis in this space group, the indexing of all data sets was tested for consistency to a reference set (PDB entry 3BC7^9^), which facilitated comparisons of these isomorphous structures. Specifically, each set was scaled to the reference using nonreindexed data and with the data reindexed as (*k*, *h*, − *l*). The indexing that gave better scaling statistics was chosen. The same reflections were marked for the test set in each case. The model from PDB entry 3BC7, without mannose or water molecules, was used as starting model for the refinement of the complex of langerin with the blood group B trisaccharide. A partially refined model of this complex, excluding all waters and sugars from the model, was used as starting model for the refinement of the other data sets. Model building and refinement were performed with Coot and PHENIX.^24,25^ Refinement included individual positional and isotropic temperature factor refinement for all atoms, except that Ca^2+^ was refined with an anisotropic temperature factor model. Refinement statistics, including the residues modeled in each case, are shown in Table 2.

### PDB accession codes

Atomic coordinates and structure factors have been deposited in the PDB, Research Collaboratory for Structural Bioinformatics§, Rutgers University (New Brunswick, NJ), with accession codes 3P5D (Man5 complex), 3P5E (Man4 complex), 3P5F (Man2 complex), 3P5G (blood group B complex), 3P5H (laminaritriose complex), and 3P5I (6SO_4_–Galβ1–4GlcNAc complex).

The following are the supplementary materials related to this article.

Supplementary Table 1 Bacterial O-specific polysaccharides on array.

## Figures and Tables

**Table 1:** Crystallographic data statistics

Data	Langerin CRD + blood group B trisaccharide	Langerin CRD + Man5	Langerin CRD + Man4	Langerin CRD + Manα1–2Man	Langerin CRD + 6SO_4_–Gal–GlcNAc	Langerin CRD + laminaritriose
Space group	*P*4_2_	*P*4_2_	*P*4_2_	*P*4_2_	*P*4_2_	*P*4_2_
Unit cell parameters *a*, *b*, *c* (Å)	79.86, 79.86, 90.17	79.34, 79.34, 89.67	79.31, 79.31, 89.58	79.78, 79.78, 90.47	79.81, 79.81, 90.74	80.11, 80.11, 90.72
Resolution (last shell) (Å)	39.93–1.60 (1.69–1.60)	44.83–1.80 (1.90–1.80)	44.79–1.70 (1.79–1.70)	39.89–1.75 (1.84–1.75)	47.92–1.80 (1.90–1.80)	36.64–1.60 (1.69–1.60)
*R*_sym_ (last shell)^a^	5.3 (24.0)	6.9 (37.4)	6.3 (40)	6.3 (24.8)	6.5 (28.1)	5.3 (28.1)
Mean ((*I*)/σ(*I*)) (last shell)	13.9 (4.8)	18.2 (5.4)	20.3 (4.9)	20.0 (7.3)	18.6 (6.5)	22.0 (6.4)
% Completeness (last shell)	100 (100)	100 (99.9)	100 (100)	100 (100)	100 (100)	99.9 (100)
Number of unique reflections	74,505	51,453	60,951	57,096	52,682	75,402
Average multiplicity (last shell)	3.8 (3.8)	7.7 (7.6)	7.6 (7.6)	7.6 (7.6)	7.7 (7.6)	7.6 (7.5)

a *R*_sym_ = ∑_*h*_∑_*i*_(|*I*_*i*_(*h*)| − |〈*I*(*h*)〉|)/∑_*h*_∑*_i_I_i_*(*h*), where *I*_*i*_(*h*) is the observed intensity, and 〈*I*(*h*)〉 is the mean intensity obtained from multiple measurements.

**Table 2:** Crystallographic refinement statistics

Data	Langerin CRD + blood group B trisaccharide	Langerin CRD + Man5	Langerin CRD + Man4	Langerin CRD + Manα1–2Man	Langerin CRD + 6SO_4_–Gal–GlcNAc	Langerin CRD + laminaritriose
*Residues in the final model*						
Copy A: protein residues	198–325	198–325	198–325	198–325	198–325	198–325
Copy A: sugars	1 Trisaccharide	2 Man	1 Man	1 Man	1 6SO_4_–Gal–GlcNAc	1 Glc
Copy B: protein residues	198–325	198–325	198–325	198–325	198–325	197–325
Copy B: sugars	1 Trisaccharide	1 Man	1 Man	2 Man	1 6SO_4_–Gal–GlcNAc	2 Glc
Copy C: protein residues	197–325	196–325	196–325	197–325	197–325	196–325
Copy C: sugars	1 Fuc	1 Man	1 Man	2 Man	1 6SO_4_–Gal–GlcNAc	1 Glc
Copy D: protein residues	197–325	197–324	197–324	197–325	197–325	197–324
Copy D: sugars	1 Fuc	1 Man	1 Man	1 Man	1 6SO_4_–Gal–GlcNAc	2 Glc
Water molecules	819	526	570	599	479	717
Number of reflections used for refinement	70,325	48,636	57,657	54,038	49,700	70,704
Reflections marked for *R*_free_	3742	2619	3099	2897	2670	3753
*R*_free_^a^	21.9	22.0	21.2	21.6	22.3	21.4
*R*_cryst_^a^	17.9	17.4	17.3	17.3	17.8	17.9
Average *B*-factor	23.5	26.7	24.6	23.1	26.2	23.7
Average *B*-factor for carbohydrate	35.8	40.2	32.8	29.9	46.2	39.0
RMSD of bond length (Å)	0.007	0.007	0.007	0.007	0.007	0.007
RMSD of bond angle (°)	1.1	1.1	1.1	1.1	1.1	1.1
Ramachandran plot						
Preferred/allowed/outliers (%)^b^	96.0/4.0/0	96.0/4.0/0.0	95.7/4.3/0.0	95.6/4.4/0.0	95.4/4.6/0.0	95.8/4.2/0.0

a *R* and *R*_free_ = ∑‖*F*_o_| − |*F*_c_‖/∑|*F*_o_|, where |*F*_o_| is the observed structure factor amplitude and |*F*_c_| is the calculated structure factor amplitude for the working and test sets, respectively.

b As defined in Coot.^25^

## Footnotes

† Available from www.functionalglycomics.org/static/consortium/resources/timelined2p.shtml

‡ www.functionalglycomics.org

3 http://www.rcsb.org/
