# Supplementary material for: Structural Basis for Langerin Recognition of Diverse Pathogen and Mammalian Glycans through a Single Binding Site
Source: J Mol Biol. 2011 Jan 28;405(4-3):1027–39. doi: 10.1016/j.jmb.2010.11.039 (PMC3065333; doi:10.1016/j.jmb.2010.11.039)
Supplement: Supplementary Table 1 — Bacterial O-specific polysaccharides on array. [file mmc1.pdf]

Supplemental Table 1. **Bacterial O-specific polysaccharides on array**
ID numbers correspond to entries in the Bacterial Carbohydrate Structure DataBase (www.glyco.ac.ru/bcsdb3).

Array	Bacterial strain	ID	Polysaccharide structure
1	*Escherichia coli* O29	20208	a-D-Glcp-(1-6)-a-D-Galp-(1-3)-+<br>| -3)-D-Gro-(1-P-6)-b-D-Glcp-(1-4)-a-L-FucpNAc-(1-3)-b-D-GlcpNAc-(1-
2	*Escherichia coli* O40	21403<br>23075	-2)-b-D-Galp-(1-4)-b-D-Manp-(1-4)-a-D-Galp-(1-3)-b-D-GlcpNAc-(1-
3	*Escherichia coli* O106	20655<br>21694	a-D-Glcp-(1-4)-+<br>| -6)-a-D-Manp-(1-2)-a-D-Manp-(1-2)-b-D-Manp-(1-3)-a-D-GlcpNAc-(1-
4	*Escherichia coli* O130	21749	Gro-(2-P-4)-b-D-GalpNAc-(1-3)-+<br>| -4)-a-D-Galp-(1-6)-b-D-Glcp-(1-3)-b-D-GalpNAc-(1-
5	*Escherichia coli* O148	21578<br>23062	-3)-a-L-Rhap-(1-3)-a-L-Rhap-(1-2)-a-D-Glcp-(1-3)-a-D-GlcpNAc-(1-
6	*Escherichia coli* O150:H6	23079<br>21743	S-lac-(2-4)-+ b-D-Glcp-(1-2)-+<br>| | -3)-b-D-GlcpNAc-(1-2)-a-L-Rhap-(1-2)-a-L-Rhap-(1-3)-a-L-Rhap-(1-3)-b-D-GlcpNAc-(1-
7	*Escherichia coli* O180/C3	10174	2)-b-D-Quip3NAc-(1-3)-b-D-Ribf-(1-4)-b-D-Galp-(1-3)-a-D-GalpNAc-(1-
8	*Proteus mirabilis* 3a, O3c	4700	L-Lys-(2-6)-a-D-GalpA-(1-4)-+<br>| -6)-b-D-GalpNAc-(1-4)-b-D-GlcpA-(1-3)-b-D-GalpNAc-(1-
9	*Proteus mirabilis* O8<br>*Proteus vulgaris* O8	4030	a-D-Galp-(1-3)-+<br>| -3)-b-D-GlcpA-(1-4)-a-L-FucpNAc-(1-3)-a-D-GlcpNAc-(1-

10 *Proteus mirabilis* O10 4664

```
a-L-AltpA-(1-3)-+
                |
       -4)-a-D-GalpNAc-(1-3)-a-D-GalpA-(1-3)-a-D-GlcpNAc-(1-
```

11 *Proteus mirabilis* O29a,29b 10129

```
a-D-GalpNAc-(1-3)-+  a-D-Glcp-(1-2)-+
                  |                 |
         -6)-b-D-GalpNAc-(1-4)-b-D-GlcpA-(1-3)-b-D-GalpNAc-(1-
```

12 *Proteus mirabilis* O50 2850

```
b-D-Glcp-(1-4)-+
               |
      -3)-b-D-GlcpA-(1-4)-b-D-GalpNAc-(1-4)-b-D-Galp-(1-3)-b-D-GlcpNAc-(1-
```

13 *Proteus mirabilis* O54a, 54b 20237

```
             D-Gro-(1-P-4)-+
                           |
-6)-a-D-GlcpNAc-(1-3)-b-D-Galp-(1-3)-a-D-GalpNAc-(1-
```

14 *Providencia alcalifaciens* O5 5945

```
-4)-b-D-Quip3NAc-(1-3)-a-D-Galp-(1-3)-b-D-Galp-(1-3)-b-D-GlcpNAc-(1-
```

15 *Providencia alcalifaciens* O19 9276

```
-2)-b-D-Fucp3NAc4Ac-(1-3)-a-D-GlcpNAc-(1-4)-a-D-Galp-(1-4)-b-D-Galp-(1-3)-b-D-GlcpNAc-(1-
```

16 *Providencia alcalifaciens* O21 2840

```
a-D-Fucp3NFo-(1-4)-+
                   |
          -3)-a-D-GalpA-(1-4)-a-D-GalpNAc-(1-4)-a-D-GalpNAc-(1-3)-b-D-GalpNAc-(1-
```

17 *Providencia rustigianni* O14 2841

```
(2S,8S)-cetLys-(2-6)-+
                     |
            -3)-a-D-GalpA-(1-4)-a-D-GalpNAc-(1-3)-a-D-GlcpNAc-(1-
```

18 *Providencia stuartii* O18 10467

```
-4)-b-D-Quip3NAc-(1-6)-a-D-GlcpNAc-(1-4)-b-D-GlcpA-(1-3)-a-D-GalpNAc-(1-
```

19 *Providencia stuartii* O44 10079

```
           b-D-GlcpA-(1-4)-+
                           |
-4)-a-D-GalpNAc-(1-3)-a-L-Fucp-(1-3)-a-D-Glcp-(1-4)-a-L-Quip-(1-3)-a-D-GlcpNAc-(1-
```

20 *Pseudomonas aeruginosa* O1 (IATS 1, FI4) 20553

```
-4)-a-D-GalpNAc-(1-4)-b-D-GlcpNAc3NAcA-(1-3)-a-D-FucpNAc-(1-3)-a-D-QuipNAc-(1-
```

21	*Pseudomonas aeruginosa* O2 (IATS 18, FI7)	20560	-4)-a-L-GulpNAc3NAmA-(1-4)-b-D-ManpNAc3NAcA-(1-3)-a-D-FucpNAc-(1-
22	*Pseudomonas aeruginosa* O2a,2b (IATS 16)	20554	-4)-b-D-ManpNAc3NAmA-(1-4)-b-D-ManpNAc3NAcA-(1-3)-b-D-FucpNAc-(1-
23	*Pseudomonas aeruginosa* O2a,2b,2e	20555	-4)-b-D-ManpNAc3NAmA-(1-4)-b-D-ManpNAc3NAcA-(1-3)-b-D-FucpNAc4Ac-(1-
24	*Pseudomonas aeruginosa* O2a,(2c) (IATS 2, FI3)	20556	-4)-b-D-ManpNAc3NAmA-(1-4)-b-L-GulpNAc3NAcA-(1-3)-a-D-FucpNAc-(1-
25	*Pseudomonas aeruginosa* O2a,2d	20557	-4)-b-D-ManpNAc3NAmA-(1-4)-b-D-ManpNAc3NAcA-(1-3)-a-D-FucpNAc-(1-
26	*Pseudomonas aeruginosa* O3a,3b	20561	(S)-3HOBut-(1-4)-+<br>| -2)-a-L-Rhap3Ac-(1-6)-a-D-GlcpNAc-(1-4)-a-L-GalpNAcA-(1-3)-b-D-QuipNAc4N-(1-
27	*Pseudomonas aeruginosa* O3a,3b,3c (IATS 3)	122939	(S)-3HOBut-(1-4)-+<br>| -2)-a-L-Rhap3Ac-(1-6)-a-D-GlcpNAc-(1-4)-a-L-GalpNAcA3Ac-(1-3)-b-D-QuipNAc4N-(1-
28	*Pseudomonas aeruginosa* O3a,3d	20563	-3)-a-L-Rhap-(1-6)-a-D-GlcpNAc-(1-4)-a-L-GalpNAcA-(1-3)-b-D-QuipNAc4NAc-(1-
29	*Pseudomonas aeruginosa* O4a,4c	20565	-2)-a-L-Rhap-(1-3)-a-L-FucpNAc-(1-3)-a-L-FucpNAc-(1-3)-a-D-FucpNAc-(1-
30	*Pseudomonas aeruginosa* O6 (FI1)	20570	-2)-a-L-Rhap-(1-4)-a-D-GalpNAcA3Ac-(1-4)-a-D-GalpNFoA-(1-3)-a-D-QuipNAc-(1-
31	*Pseudomonas aeruginosa* O6a (IATS 6)	20566	-3)-a-L-Rhap-(1-4)-a-D-GalpNAcA3Ac-(1-4)-a-D-GalpNFoA-(1-3)-a-D-QuipNAc-(1-
32	*Pseudomonas aeruginosa* O6a,6c	20568	-3)-a-L-Rhap-(1-4)-a-D-GalpNAcA-(1-4)-a-D-GalpNFoA-(1-3)-a-D-QuipNAc-(1-
33	*Pseudomonas aeruginosa* O13a,13b (IATS 13)	123031	-2)-a-L-Rhap-(1-3)-a-L-Rhap-(1-4)-a-D-GalpNAcA3Ac-(1-3)-b-D-QuipNAc-(1-

34 *Pseudomonas aeruginosa* O10a,10b (IATS 10) — 20576

```
-3)-a-L-Rhap2Ac-(1-4)-a-L-GalpNAcA-(1-3)-a-D-QuipNAc-(1-
```

35 *Pseudomonas aeruginosa* O10a,10c (IATS 19) — 20577

```
-3)-a-L-Rhap-(1-4)-a-L-GalpNAcA-(1-3)-a-D-QuipNAc-(1-
```

36 *Pseudomonas aeruginosa* O11a,11b (IATS 11) — 20578

```
-2)-b-L-Glcp-(1-3)-a-L-FucpNAc-(1-3)-b-D-FucpNAc-(1-
```

37 *Pseudomonas aeruginosa* O12 (IATS 12) — 20579

```
-8)-a-8eLegp5Ac7Ac-(2-3)-a-L-FucpNAm-(1-3)-a-D-QuipNAc-(1-
```

38 *Pseudomonas aeruginosa* O13a,13b (IATS 13) — 20580

```
-2)-a-L-Rhap-(1-3)-a-L-Rhap-(1-4)-a-D-GalpNAc3AcA-(1-3)-b-D-QuipNAc-(1-
```

39 *Pseudomonas aeruginosa* O13a,13c (IATS 14) — 20581

```
a-D-Glcp-(1-3)-+
               |
      -2)-a-L-Rhap-(1-3)-a-L-Rhap-(1-4)-a-D-GalpNAcA-(1-3)-b-D-QuipNAc-(1-
```

40 *Pseudomonas aeruginosa* O14 (IATS 17) — 20582

```
-4)-a-L-Rhap-(1-3)-b-D-ManpNAc-(1-
```

41 *Pseudomonas aeruginosa* O15 (IATS 15) — 20583

```
-2)-b-D-Ribf-(1-3)-a-D-GalpNAc-(1-
```

42 *Shigella boydii* type 2 — 114007

```
b-D-Galf-(1-3)-+
               |
      -2)-a-L-Rhap-(1-2)-a-L-Rhap-(1-2)-b-D-Ribf-(1-4)-a-D-GalpA-(1-3)-a-D-GlcpNAc-(1-
```

43 *Shigella boydii* type 4 — 108649

```
                         a-D-Glcp-(1-2)-+
                                        |
-3)-a-L-Rhap-(1-4)-b-D-GlcpA-(1-3)-b-L-Rhap-(1-4)-b-D-GlcpNAc-(1-
```

44 *Shigella boydii* type 10 — 10148

```
b-D-Ribf-(1-4)-b-D-GlcpA-(1-4)-+
                               |
                      -3)-a-D-Galp-(1-6)-a-D-Manp-(1-2)-a-D-Manp-(1-3)-b-D-GalpNAc-(1-
```

45 *Shigella dysenteriae* type 3 — 20016

```
(R)-lac-(2-4)-b-D-Glcp-(1-6)-a-D-Glcp-(1-4)-+
                                             |
                                    -3)-b-D-Galp-(1-6)-b-D-Galf-(1-3)-b-D-GalpNAc-(1-
```

46 *Shigella dysenteriae* type 8 — 108662

```
          b-D-GlcpNAc-(1-4)-b-D-Glcp-(1-4)-+
                                           |
-4)-b-D-GlcpA-(1-3)-b-D-GalpNAc-(1-3)-b-D-GalpNAc-(1-
```

47 *Shigella dysenteriae* type 11 — 23077

```
a-D-Glcp-(1-6)-a-D-Galp-(2-Ac ~25%)-(1-3)-+
                                          |
    -3)-D-Gro-(3-P-6)-b-D-Glcp-(1-4)-a-L-FucpNAc-(1-3)-b-D-GlcpNAc-(1-
```

48 *Shigella dysenteriae* type 13 — 23079

```
(S)-lac-(2-4)-+                  b-D-Glcp-(1-2)-+
              |                                 |
     -3)-b-D-GlcpNAc-(1-2)-a-L-Rhap-(1-3)-a-L-Rhap-(1-3)-b-D-GlcpNAc-(1-
```

Abbreviations: Am, acetimidoyl; cetLys, N^{e}-(1-carboxyethyl)lysine; Fo, formyl; lac, 1-carboxyethyl (a lactic acid residue); 3-HOBut, 3-hydroxybutanoyl; 8eLeg, 5,7-diamino-3,5,7,9-tetradeoxy-L-glycero-D-manno-non-2-ulosonic (8-epilegionaminic) acid.
